# Associations between body mass index, weight control concerns and behaviors, and eating disorder symptoms among non-clinical Chinese adolescents

**DOI:** 10.1186/1471-2458-10-314

**Published:** 2010-06-06

**Authors:** Yiou Fan, Yanping Li, Ailing Liu, Xiaoqi Hu, Guansheng Ma, Guifa Xu

**Affiliations:** 1 Department of Nutrition and Food Hygiene, School of Public Health, West Campus of Shandong University, No.44 Wenhuaxi Road, Ji' nan, Shandong 250012, PR China; 2 Institute of Nutrition and Food Safety, China Center for Disease Control and Prevention, No.29, Nanwei Road, Xuanwu District, Beijing, 100050, PR China; 3 Institute of Toxicology, Shandong Center for Disease Control and Prevention, No.72 Jingshi Road, Lixia District, Ji' nan, Shandong 250014, PR China

## Abstract

**Background:**

Previous research with adolescents has shown associations of body weight, weight control concerns and behaviors with eating disorder symptoms, but it is unclear whether these associations are direct or whether a mediating effect exists. This study was conducted to investigate the prevalence of overweight and obesity, weight control concerns and behaviors, and eating disorder symptoms and to examine the mediating function of weight control concerns and behaviors on the relationship between body mass index (BMI) and eating disorder symptoms among non-clinical adolescents in China.

**Methods:**

A cross-sectional survey among 2019 adolescent girls and 1525 adolescent boys in the 7th, 8th, 10th and 11th grades from seven cities in China was conducted. Information on weight control concerns and behaviors, and eating disorder symptoms (Eating Disorder Inventory-3) were collected from the adolescents using a self-administrated questionnaire.

**Results:**

Weight control concerns and behaviors, and eating disorder symptoms were prevalent among the study population. A high proportion of adolescents scored at or above the threshold on the eating disorder inventory (EDI) subscale such as bulimia, interoceptive deficits, perfectionism, and maturity fears, which indicated eating disorder symptoms. High BMI was significantly associated with high score of drive for thinness, body dissatisfaction, bulimia, low self-esteem, interceptive deficits and maturity fears, so do perceived body weight status. Almost all weight control concerns and behaviors we investigated were significantly associated with high EDI subscale scores. When weight control concerns were added to the model, as shown in the model, the association between BMI and tendency of drive to thinness and bulimia was attenuated but still kept significant. The association between BMI and body dissatisfaction were no further significant. The association of BMI and drive for thinness, body dissatisfaction and bulimia was considerably weaker than when weight control behaviors were not included.

**Conclusions:**

Weight control concerns and behaviors may be mediators of the association between BMI and eating disorder symptoms. Interpretation of these weight control problems is crucial to develop culturally appropriate educational and intervention programs for adolescents.

## Background

Eating disorders, such as anorexia, bulimia, and binge eating disorder, are associated with extreme emotions, attitudes, and behaviors, as well as physical problems that can have life-threatening consequences. These consequences are affecting adolescents with increasing frequency. Adolescent obesity is one of the risk factors for the development and maintenance of eating disorder symptoms[1-5]. One possible reason for this may be that overweight adolescents show greater concern about body image and a greater tendency to perform dietary restraint than their counterparts with normal weight. A number of studies have identified the association of increased risk for eating disorder symptoms with elevated weight and shape concerns, and unhealthy weight loss behaviors in overweight adolescents[1,6-10].

A recent review[11] on the epidemiology of eating disorders in Western countries indicated that the prevalence rates for anorexia nervosa ranged from 0.1% to 5.7% in females, and for bulimia nervosa from 0% to 2.1% in males and from 0.3% to 7.3% in females. The prevalence in non-Western countries for bulimia nervosa ranged from 0.46% to 3.2% in female subjects. Several school-based studies in China have reported the prevalence of eating disorders varied from 1.3% to 5.21% among females aged 15 to 24 years old[12-16]. However, due to the lack of a national epidemiological investigation in China, these data are not representative of the entire population.

Another public health concern related to weight is the high prevalence of weight control concerns and behaviors among adolescents. These concerns and practices are on a continuum ranging from healthy to unhealthy. Many overweight and obese adolescents display elevated risk for weight/shape concerns and dieting attempts which, in turn, are associated with disturbance in eating habits. Longitudinal studies have found that weight and shape concern and weight control behaviors are potent predictors for future onset of full- and sub-threshold anorexia nervosa and bulimia nervosa[17-20]. Population-based studies showed that half of adolescent girls and one third of adolescent boys reported unhealthy weight control behaviors such as dieting and excessive exercise, and 7-12% of adolescent girls and 3-7% of adolescent boys tried to lose weight by extreme behaviors such as vomiting, taking diet pills, or laxatives[21-26]. To date, little is known about the prevalence of weight control concerns and behaviors in Chinese population.

Previous studies of associations between body weight, weight control concerns and practices, and eating disorder symptoms have included measures of BMI, weight control concerns and practices or eating disorder symptoms but, to this point, all three measures have not been systematically investigated within a single study[1,6,25,27-33]. Therefore, the independent effects of BMI and weight control concerns and practices on eating disorder symptoms are not clear. BMI and weight control concerns and practices may each have a direct or indirect association with eating disorder symptoms.

Based on the evidence that elevated adiposity is theorized to contribute to weight control concerns and behaviors among adolescents [34-39] and that weight/shape concern and weight control behaviors increases risk for the onset and maintenance of eating disorders symptoms[7,28,31,40], we hypothesized that weight control concerns and behaviors may mediate the association between BMI and eating disorder symptoms. Given the rather limited literature concerning the role of weight control concerns and behaviors among non-clinical Chinese adolescents, the current study tested this hypothesis using a national representative survey of adolescents.

The purpose of this study are: firstly, to investigate the prevalence of overweight or obesity, weight control concerns and behaviors, and eating disorder symptoms in a population of non-clinical Chinese adolescents; secondly, to study whether BMI category, or weight control concerns and behaviors significantly contribute to eating disorder symptoms either individually, or in concert; and lastly, to determine if weight control concerns and behaviors will mediate the association of BMI category and eating disorder symptoms.

## Methods

### Study Population and Design

We used a random cluster-sampling survey design, in which schools were considered as clusters. A total of 56 school classes containing the 7th, 8th, 10th, and 11th grade students were selected from 7 cities in China. Two high schools in middle-class to upper middle-class urban areas were selected randomly from each city. Within each school, two classes from each grade were randomly selected.

The Ethical Review Committee at the National Institute for Nutrition and Food Safety, Chinese Center for Disease Control and Prevention provided the ethical approval. Written informed consent was obtained from both parents and subjects.

### Instrument design

The Student Questionnaire and Parent Questionnaire were designed for paper and pencil collection. Student questionnaire contained items to gather information on weight control concerns and behaviors. Information on the socio-economic status was collected using questionnaire from parents.

### Measures

#### 1. BMI Category

Weight and height were measured by trained investigators following a standardized protocol. At each examination, height to 0.1 cm and weight to 0.1 kg were measured. Body mass index (BMI, kg/m^2^) was calculated by dividing the weight (kg) by the height (m) squared. Subjects were classified into four groups (underweight, normal weight, overweight and obesity) according to the age-, sex-specific BMI reference recommended by the Working Group on Obesity in China (WGOC)[41]. Each child was evaluated separately to decide whether he or she falls in the underweight, normal weight, overweight or obese category in comparison with the standard (WGOC) of his/hers own age and gender.

#### 2. Sociodemographic Variables

Sociodemographic variables including age, sex, and parental educational level, employment status of the parents, and family income.

#### 3. Eating Disorder Inventory-3 (EDI-3) Items

The EDI-3 is a widely used self-report measure of psychological traits or constructs, and has previously been shown to be clinically relevant in individuals with eating disordes [42]. This questionnaire has good internal consistency: between 0.84 and 0.92 for each scale[43]. Leung et al has reported the psychometric properties of the EDI among Chinese adolescent girls in Hong Kong[44]. Findings provide empirical support to the transcultural validity of the EDI, which indicated the questionnaire had good reliability and validity among adolescent girls in Hong Kong. After getting the permission from the copyright owner of EDI (Psychological Assessment Resources, Inc), the EDI-3 was translated into Mandarin Chinese.

There was 91 items of EDI-3. For each item, participants were required to indicate the frequency of the concern or behavior on a six-point scale ranging from "always" to "never". For the positive items, the point was 0,0,1,2,3,4, while for the negative item, the point was 4,3,2,1,0,0 for "always", "usually", "often", "sometimes", "rarely" and "never", respectively. Higher scores indicating higher likelihood of eating disorders.

In this study, seven subscales of EDI-3 were used, among which drive for thinness (DT), bulimia (B), and body dissatisfaction (BD) are items related to the diagnostic criteria of eating disorder symptoms, and subscales of low self-esteem (LSE), interoceptive deficits (ID), perfectionism (P), and maturity fears (MF) are items for evaluating psychopathologic trait commonly seen in eating disorders. On comparing the positive cases we used seven cut-off points, DT raw score ≥ 16, B raw score ≥ 5, BD raw score ≥ 22, LSE raw score ≥ 9, ID raw score ≥ 11, P raw score ≥ 10, and MF raw score ≥ 6 according to the options found in the EDI-3 manual.

The EDI Cronbach's alpha was 0.78 in the current study, while internal consistency reliability estimates for the 7 subscales of the EDI were 0.73, 0.75, 0.73, 0.77, 0.76, 0.77 and 0.76 for drive for thinness, body dissatisfaction, bulimia, perfectionism, maturity fears, low self-esteem and interceptive deficits, respectively.

#### 4. Weight Control Concerns and Behaviors

The information on weight control concerns and behaviors were investigated by a self-administrated questionnaire. There were 8 items of weight control concerns and of weight control behavior, respectively. The question for weight control concerns are "whether you ever think about of weight control by next ways" while the question for weight control behavior was "whether you did do the following things in aims of weight control". The 8 items of weight control ways were: "avoid ate sweet foods", "avoid ate fatty foods", "skip staple food", "excessive exercise", "diet pills, foods or tea (the special ones somehow called weight loss pill, food or tea)", "surgery", "Smoking", "Dieting". Two items are not totally matched in concerns and behavior. The first one is "Exercise". We asked whether they considered of "exercise" and whether they did "excessive exercise" before, which means concern of exercise and behavior of excessive exercise. The second one is "Diet pill, food and tea". We asked only one question about considering "Diet pill, food and tea", but two questions for behavior (one is "try diet pills before, the other is try "diet food and tea before"). Weight control concerns and behaviors were based on YES-or-NO answers to questions assessing the presence or absence of the concerns or behaviors of weight control.

#### 5. Weight perception

Weight perceptions were assessed by the question "What do you think about your own body weight?" The response categories were: (1) very underweight; (2) slightly underweight; (3) about the right weight; (4) slightly overweight; (5) very overweight. In the data analysis, these responses were recoded into four categories: underweight, normal weight, overweight and obesity.

### Statistical Analysis

Adjusted means and standard error of the seven subscale of Eating Disorder Inventory were calculated and compared after control for the cluster effect of classes and the socioeconomic status. Binary terms for each EDI subscale score were created on the basis of the recommended cutoff points provided in the EDI manual book as an indication of a possible ED. Binary terms were created for each of the items on weight control concerns and behaviors. Descriptive analysis (means and percentages) was conducted to examine the sex differences in the study, including EDI scales, weight control concerns and behaviors.

To test for the mediating role of weight control concerns and behaviors on the relationship between BMI category and eating disorder symptoms, the method proposed by Baron and Kenny[45] was used. The following conditions were tested: (1st condition) both weight control concerns and behaviors are related to BMI; (2nd condition) BMI is related to eating disorder symptoms (EDI); and (3rd condition) the significant relationship of BMI and eating disorder symptoms when perceived weight, other weight control concerns and weight control behaviors are considered in the analysis. Perceived weight was used as an ordered, three-category response variable (under weight, normal weight, overweight/obesity).

The data were tested with a mixed regression model after controlling the clustering effect of students in classes. Demographic factors were adjusted in the analyses. Stepwise procedure was implied to select the items of weight control concerns or behaviors that jointly associated with EDI subscales, together with BMI and other relative factors. The selected items and BMI together were put in the mixed model as fixed effects on EDI subscales while the clustering of students in class was treated as random effect.

## Results

### Characteristics of the subjects

A total of 3685 student questionnaires were released. The response rate was 100%, among which 3544 were included for the data analysis based on the number of returned valid questionnaires. A total of 3685 parent questionnaires was released, the response rate was 97% (n = 3573). The percentage of valid questionnaires was 98% (n = 3503), including those that did not answer two or more questions.

Among 3544 students, 1525 (43.0%) were male and 2019 (57.0%) were female, with an age range between 14 and 18 years old (mean age of 15.6 ± 0.7 years). Among these adolescents, 5.1% of boys and 5.8% of girls were categorized as "underweight", 72.5% of boys and 85.8% of girls were categorized as "normal weight", 14.8% of boys and 6.2% of girls were categorized as overweight, and 7.5% of boys and 2.1% of girls were categorized as "obese". Among girls, perceived weight status was in accordance with actual weight status. Among boys, the order of the distributions slightly differs between perceived weight and BMI category. The characteristics of the students are shown in Table 1. No significant difference was found in age between boys and girls. Significantly more boys were classified as overweight or obese than girls. The parents' educational level, family income level, and parents' employment status of boys were higher than those of girls.

**Table 1:** Sample Characteristics

Characteristics	Boys(n = 1525)	Girls (n = 2019)	Total (n = 3544)	t or χ^2^
Age (years)				
	15.6 ± 0.6	15.6 ± 0.8	15.6 ± 0.7	-1.39
Weight Status^1^				
Underweight	5.1%	5.8%	5.5%	142.91***
Normal weight	72.5%	85.8%	82.8%	
Overweight	14.8%	6.2%	10.5%	
Obese	7.5%	2.1%	4.8%	
Perceived body weight				
Underweight	28.1%	16.9%	21.7%	85.47***
Normal weight	45.8%	46.6%	46.3%	
Overweight	21.6%	32.1%	27.6%	
Obese	3.8%	4.0%	3.9%	
Father's Educational Level^2^				
Low	24.7%	28.5%	26.8%	19.94***
Middle	53.9%	55.7%	31.6%	
High	21.4%	15.8%	18.2%	
Mother's Educational Level^2^				
Low	30.4%	34.3%	32.6%	24.12***
Middle	53.5%	55.1%	54.4%	
High	16.0%	10.6%	12.9%	
Family's Income^3^				
Low	21.7%	29.1%	25.9%	50.12***
Middle	51.9%	53.2%	52.7%	
High	26.4%	17.6%	21.4%	
Father employment status^4^				
Yes	78.3%	61.1%	69.7%	0.11
No	21.7%	38.9%	30.3%	
Mother employment status^4^				
Yes	68.3%	64.1%	66.2%	6.87**
No	31.7%	35.9%	33.8%	

^1 ^Defined according to the Chinese reference developed by the Working Group for Obesity in China (WGOC).

^2 ^Educational level: Low: lower than junior middle school; Middle: high middle school; High: college/university or above

^3 ^Family's income: Low: < 2000 Yuan/Month; Middle: 2000-6000 Yuan/Month; High: ≥6000 Yuan/Month

^4 ^Parents' employment status: yes: have a job; no: housework, job-waiting/laid off, and retired.

Significant difference between boys and girls, *t*-test for means or χ^2^-test for proportion, ** *P *< 0.01, *** *P *< 0.001.

### Means for EDI Subscales and Prevalence Estimates for Weight Control Concerns and Behaviors by Sex

Table 2 shows the mean scores for EDI subscales and the percentage distributions of weight control concerns and behaviors. Girls show significantly higher score of drive for thinness, body dissatisfaction, bulimia, low self-esteem, interceptive deficits and maturity fears.

**Table 2:** Means (Standard Deviations) for EDI-3 Subscales and Percentage for Weight-Related Concerns and Behaviors among Chinese Adolescents

	Boys (n = 1525)	Girls (n = 2019)	Total (n = 3544)	t or χ^2^
**EDI subscale**				
B	1.7 ± 2.3	3.4 ± 2.5	2.7 ± 2.6	-19.96***
B ≥ 5	14.0%	34.7%	25.8%	194.71***
ID	8.9 ± 3.2	9.9 ± 3.3	9.5 ± 3.3	-8.83***
ID ≥ 11	28.0%	42.4%	36.2%	77.46***
P	5.6 ± 2.6	5.8 ± 2.3	5.7 ± 2.4	-2.88**
P ≥ 10	5.1%	5.0%	5.0%	0.048
MF	3.9 ± 2.2	4.4 ± 2.0	4.2 ± 2.1	-7.41***
MF ≥ 6	22.7%	28.7%	26.1%	16.16***
DT	2.3 ± 2.9	4.2 ± 3.1	3.4 ± 3.2	-18.94***
BD	2.1 ± 2.5	2.5 ± 2.5	2.3 ± 2.5	-4.81***
LSE	1.0 ± 1.5	1.3 ± 1.6	1.2 ± 1.5	-4.99***
**Weight control concerns (Ever considered of it)**				
Avoid ate sweet foods	32.9%	47.3%	35.0%	74.29***
Avoid ate fatty foods	46.4%	71.8%	71.8%	235.13***
Skip staple food	7.0%	18.5%	13.6%	98.07***
Exercise	50.4%	71.7%	62.5%	167.33***
Diet pills, foods or tea	5.3%	17.2%	12.1%	116.29***
Surgery	2.0%	4.5%	3.5%	16.05***
Smoking	1.5%	1.3%	1.4%	0.31
Dieting	9.4%	30.0%	21.1%	222.02***
**Weight control behaviors**				
Avoid ate sweet foods	27.5%	36.7%	32.7%	33.44***
Avoid ate fatty foods	34.1%	56.6%	46.9%	174.55***
Skip staple food	5.9%	15.9%	11.6%	85.36***
Excessive exercise	44.1%	69.5%	58.6%	232.26***
Diet pills	4.1%	15.9%	10.8%	127.62***
Diet foods or tea	2.4%	6.2%	4.6%	28.30***
Surgery	1.7%	4.4%	3.2%	19.81***
Smoking	1.1%	1.0%	1.1%	0.04
Dieting	7.0%	27.2%	18.5%	235.05***

Abbreviations: DT, drive for thinness; BD, body dissatisfaction; B, bulimia; LSE, low self-esteem; ID, interoceptive deficits; P, perfectionism; MF, maturity fears.

Data are given as percentage or mean ± standard deviation (after adjusted the clustering effect of school, gender, parent's education and careers as well as family income level).

Significant difference between boys and girls, *t*-test for means or χ^2^-test for proportion, ** *P *< 0.01, *** *P *< 0.001.

Girls were also more likely than boys to have thoughts of avoiding eating sweet and/or fatty foods, skipping staple food, exercising, trying diet pills and somehow called diet food or diet tea, even surgery and dieting. More than 17% girls ever thought of skipping staple food or try diet pills, and 15.9% did do it, which was significantly higher than that among boys. The proportion of reported dieting among girls was significantly higher than that among boys (27.2% vs. 7.0%). Only a few people considered of control body weight by smoking.

### Association between BMI, perceive body weight, weight control concerns and behaviors with eating disorder symptoms

A significant positive association was identified between BMI and score of drive for thinness, body dissatisfaction, bulimia, low self-esteem, interceptive deficits, maturity fears, and perceived body weight status as well.

Almost all weight control concerns and behaviors were significantly associated with high EDI subscale scores. The more they concerned their body weight control, the higher the likelihood of their eating disorder symptoms. Similar trend was found of the association between weight control behavior and EDI subscales. Presenting of weight control behaviors was significantly associated with higher likelihood of eating disorder symptoms (Table 3).

**Table 3:** Association between BMI, perceived body weight, weight control concerns and behaviors with the scores of EDI subscales

	DT	BD	B
	*β*	*β*	*β*
**BMI**	0.2928***	0.06184***	0.2724***
**Perceived weight**	1.63***	0.2533***	1.3814***
**Weight control concerns (Ever considered of it)**			
Avoid ate sweet foods	2.6918***	0.3544***	1.4333***
Avoid ate fatty foods	2.8307***	0.4293***	1.7411***
Skip staple food	3.3805***	1.6154***	2.0271***
Exercise	2.6863***	0.4730***	1.8936***
Diet pills, foods or tea	3.0375***	1.6823***	1.8895***
Surgery	3.0761***	2.4673***	2.0619***
Smoking	3.0181***	3.6848***	3.1996***
Dieting	3.7800***	1.4726***	2.1925***
**Weight control behaviors**			
Avoid ate sweet foods	2.2070***	0.1054	1.1227***
Avoid ate fatty foods	2.5170***	0.2261*	1.4029***
Skip staple food	3.3720***	1.7260***	2.0464***
Excessive exercise	2.8508***	0.3547***	1.9823***
Diet pills	3.0611***	1.6111***	1.8199***
Diet foods or tea	3.1816***	1.9024***	1.8874***
Surgery	2.9332***	2.2534***	1.8977***
Smoking	2.9677***	3.2909***	2.9780***
Dieting	3.7627***	1.4649***	2.1189***

* *P *< 0.05, *** *P *< 0.001.

^1 ^β: linear longitudinal regression coefficient. BMI: body mass index.

^2 ^Mixed Model, other variables included in the model were gender, clustering effect of class, gender, parent's education and careers, as well as family income level.

### Adjusted Odds Ratios for the Association between Body Mass Index (BMI) and Weight Control Concerns and Behaviors

Table 4 displays the association between BMI and weight control concerns and behaviors, after controlling for covariates. Compared with normal weight adolescents, overweight and obese adolescents not only were more likely to consider but also did try different ways of weight control including avoid sweet/fatty foods, skip staple food, exercise, diet pills and dieting. Obese adolescents were also more likely to consider of surgery compared with their counterparts with normal weight.

**Table 4:** Adjusted Odds Ratios (95% confidential intervals) of presenting weight control concerns and behaviors among underweight, overweight and obese adolescents compared to their normal weight counterparts

	Normal weight	Underweight	Overweight	Obesity
**Weight control concerns(Ever considered of it)**				
Avoid ate sweet foods	1.0	0.34 (0.24-0.49)	1.73 (1.37-2.18)	1.84 (1.32-2.58)
Avoid ate fatty foods	1.0	0.38 (0.28-0.52)	1.79 (1.40-2.29)	2.88 (1.96-4.23)
Skip staple food	1.0	0.39 (0.21-0.73)	1.77 (1.29-2.42)	1.81 (1.13-2.89)
Exercise	1.0	0.28 (0.20-0.38)	2.68 (2.05-3.50)	3.68 (2.42-5.60)
Diet pills, foods or tea	1.0	0.56 (0.32-0.99)	1.45 (1.02-2.05)	2.48 (1.56-3.95)
Surgery	1.0	0.60 (0.22-1.64)	1.55 (0.88-2.74)	2.99 (1.53-5.84)
Smoking	1.0	1.12 (0.34-3.69)	0.78 (0.27-2.21)	1.32 (0.40-4.39)
Dieting	1.0	0.23 (0.13-0.42)	1.82 (1.38-2.41)	3.40 (2.31-5.01)
**Weight control behaviors**				
Avoid ate sweet foods	1.0	0.44 (0.30-0.64)	1.48 (1.17-1.87)	1.77 (1.26-2.48)
Avoid ate fatty foods	1.0	0.46 (0.34-0.64)	1.35 (1.07-1.70)	1.55 (1.11-2.16)
Skip staple food	1.0	0.49 (0.26-0.91)	1.73 (1.22-2.43)	2.09 (1.29-3.39)
Excessive exercise	1.0	0.24 (0.17-0.33)	2.94 (2.26-3.83)	3.71 (2.50-5.51)
Diet pills	1.0	0.59 (0.33-1.06)	1.46 (1.00-2.13)	3.05 (1.90-4.90)
Diet foods or tea	1.0	0.68 (0.29-1.56)	1.66 (1.01-2.73)	1.19 (0.51-2.77)
Surgery	1.0	0.69 (0.25-1.92)	1.62 (0.89-2.98)	3.24 (1.61-6.53)
Smoking	1.0	0.49 (0.07-3.63)	1.10 (0.38-3.15)	1.23 (0.29-5.20)
Dieting	1.0	0.21 (0.11-0.42)	1.98 (1.47-2.67)	3.36 (2.22-5.09)

^1 ^Abbreviations: CI, confidence interval.

^2 ^The odds ratios (95% CI) shown in the table are from the logistic regression analyses, statistically controlling for the effects of sex, age, parental educational level, employment status of the mother or father, and family income. Normal weight adolescents are used as the reference group.

### Joint association of BMI, perceive body weight, weight control concerns and behaviors with eating disorder symptoms

The initial analyses appeared to show similar patterns of odds ratios among boys and girls for the association between BMI and EDI subscales, with or without weight control concerns and behaviors. Thus, sex was seen as a potential confounding factor in the following mixed models.

Table 5 shows multivariate linear regression analyses of Models 1-4. Model 1 shows the association between BMI and EDI subscales without perceived body weight, weight control concerns and behaviors. Model 2 further controls for perceived weight from Model 1, Model 3 further controls for weight control concerns from Model 1, Model 4 further controls for weight control behaviors from Model 1. Model 1 and 2 showed that perceived body weight totally mediated the association between BMI and the score of body dissatisfaction, the associations between BMI and the score of drive for thinness or bulimia were partially mediated by perceived body weight.

**Table 5:** Joint association between body mass index (BMI), weight control concerns and behaviors with EDI subscales among Chinese adolescents

	EDI subscales		
	
	DT	BD	B
**Model 1^†^**			
BMI	0.2928**	0.0618**	0.2724**
**Model 2^†^**			
BMI	0.0792**	0.0315	0.1015**
Perceive weight	1.4786**	0.1939**	1.1858**
**Model 3^†^**			
BMI	0.1254**	0.0269	0.1762**
Avoid ate sweet foods	1.1527**	-	0.3549**
Avoid ate fatty foods	0.9285**	-	0.5393**
Skip staple food	0.9774**	0.6856**	0.5977**
Exercise	0.9846**	-	0.8724**
Diet pills, foods or tea	0.8947**	0.7668**	0.5325**
Surgery	-	0.7599**	-
Smoking	-	2.0807**	1.6087**
Dieting	2.0024	0.7268**	0.9470**
**Model 4^†^**			
BMI	0.1302**	0.0323*	0.1768**
Avoid ate sweet foods	0.7286**	-0.2328**	0.1552
Avoid ate fatty foods	0.9854**	-	0.4567**
Skip staple food	1.1126**	0.9573**	0.7503**
Excessive exercise	1.4194**	-	1.1273**
Diet pills	0.6495**	0.5403**	0.3858**
Diet foods or tea	0.7217**	0.6529**	0.3694
Surgery	-	0.7169**	
Smoking	-	1.7958**	1.4718**
Dieting	2.0300**	0.7062**	0.8978**

* *P *< 0.05, ** *P *< 0.01, Mixed Model.

^† ^Model 1 includes BMI, gender, clustering effect of class, gender, parent's education and careers, as well as family income level.

Model 2 further control perceived body weight from model 1.

Model 3 further control weight control concerns from model 1.

Model 4 further control weight control behaviors from model 1.

^**‡ **^**"-" **means that the variable was excluded from the model by stepwise procedure.

When weight control concerns were added to the model, as shown in model 3, the association between BMI and tendency of drive to thinness and bulimia were attenuated but still kept significant. The association between BMI and body dissatisfaction were no further significant. Thus, the third condition is accepted. Model 4 also showed that the association of BMI and drive for thinness, body dissatisfaction and bulimia was considerably weaker than when weight control behaviors were not included. Thus, the third condition is also accepted for weight control behaviors.

## Discussion

The main aim of present study was to investigate whether weight control concerns and behaviors mediate the association between BMI and eating disorder symptoms. In terms of the current sample, without adjusting for weight control concerns and behaviors, BMI was associated significantly with eating disorder symptoms among all adolescents. This direct association previously has been reported by numerous studies[1,3,46-48]. Our findings also suggest an association of weight control concerns and weight control behaviors with eating disorder symptoms; it appears likely that overweight or obesity concurrent with weight control concerns and weight control behaviors may compound the likelihood for the eating disorder symptoms. The developmental path leading to eating disorders among adolescents may proceed from increasing body size, to increasing weight control concerns, then to resorting to the use of unhealthy or extreme weight control behaviors, and, ultimately, to increased risk for numerous negative psychosocial sequelae [49].

Our findings demonstrate that overweight or obese Chinese adolescents were more likely to exhibit or perform weight control concerns or behaviors compared to their normal-weight peers. This finding is consistent with related research that has shown that adolescents who have extreme body size are at increased risk for unhealthy weight control concerns and behaviors[26,50]. Adolescents who feel overweight are more likely to be actively trying to lose weight and may be at risk for using harmful weight control behaviors. Overweight girls appear to be at greater risk for participating in unhealthy or extreme weight control practices than overweight boys, reflecting the greater shape and weight concerns observed among overweight girls[25,26].

Our hypothesis that the relationship between BMI and eating disorder symptoms is mediated by weight control concerns and behaviors was generally supported, based on the eight weight control concerns and behaviors that we investigated and included in current analysis. Following the guidelines for mediation analysis suggested by Baron and Kenny[45], we found that weight control concerns and behaviors met all 3 specified conditions for a full mediator in the association of BMI and interceptive deficits and maturity fears. While the conditions for partial mediation were satisfied in the case of weight control concerns behaviors on the association between BMI and drive to thinness and bulimia. The association between BMI and body dissatisfaction was fully mediated by the weight control controls and partially by weight control behaviors. This result suggests that weight-related concerns and behaviors are not only an independent indicator of eating disorder symptoms, but also serve as the mediator between BMI and eating disorder symptoms. Thus, prevention strategies would be very useful if they focus on the deleterious effects of these mediator variables on eating disorder symptoms.

Weight control concerns are necessary for the development of eating disorder symptoms, and these concerns may be a vital step on the pathway to an eating disorder[18]. It has been suggested that weight or shape concerns would be seen as a core diagnostic features of all eating disorder symptoms[51]. Recently conducted research suggested that weight and shape concerns may play a particularly key role in the cognitive models of anorexia nervosa and bulimia nervosa[52]. Nevertheless, the fact that there are still some effects of BMI on drive for thinness and bulimia after adjusting for the mediating effects of weight control concerns and behaviors implies that BMI remains a strong indicator of these eating disorder symptoms even after the mediating effects are taken into account.

We extend the work of others who have identified relationships between BMI and eating disorder symptoms that a considerable proportion of the drive to thinness, body dissatisfaction and bulimia among adolescents were attributable to their weight control concerns and behaviors. We did find that the adolescents who present early onset of an eating disorder symptom were significantly associated with more concerns of their body weight and did show more likelihood of unhealthy weight control behaviors including dieting and excessive exercise.

The strength of present study is the large and representative sample of Chinese adolescents. The joint information of body weight, weight control concerns and behaviors, and eating disorder symptoms in one study was also a strength of present study. Chinese adolescents were thought to be more tolerance of excess body weight and were assumed of less psychological disorders. Present study indicated that it may not be the truth. Weight control concerns and behaviors, and eating disorder symptoms were prevalent among the study population. A high proportion of adolescents scored at or above the threshold on the eating disorder inventory (EDI) subscale such as bulimia, interoceptive deficits, perfectionism, and maturity fears, which indicated the potential risk of eating disorders. These phenomena should be pay attention to by both clinical and public health worker in China.

We have to clarify several limitation of present study. The cross-sectional nature of the present study is indeed a limitation. This cross-sectional nature only allows drawing the conclusions with regarding the association between BMI, weight control concerns and behaviors, and eating disorder symptoms. It did limit the ability to conclude whether weight control concerns and behaviors are just on the pathway from BMI to eating disorder symptoms. Future prospective study and intervention studies are needed to confirm the present finding that weight control concerns and behaviors may be partially or fully mediate the association between BMI and eating disorder symptoms. Another limitation of this study is that the EDI-3 questionnaire was developed for Western population. To our knowledge, the EDI-3 rating scale has not been formally validated in Chinese populations. Leung et al.[44] have validated the psychometric properties of the EDI-2 among Chinese adolescent girls in Hong Kong. The 7 subscales used in the current study are agreed with EDI-2 subscales. The self-report measures used in the present study may lead to underreporting or to underestimation of eating disorder symptoms. No validated weight control concerns and behaviors questionnaire was also one limitation of present study, which may have missed some real important issues of weight control concerns and behaviors. However, even based on current 8 items, we already found the significant joint association of BMI and weight related concerns and behaviors with the eating disorder symptoms.

## Conclusions

The results of this study provide new information on the nature of the mediating effect of weight control concerns and behaviors on the relationship between BMI and eating disorder symptoms and support the inclusion of weight control concerns and behaviors as variables in models aimed at explaining risks of eating disorder symptoms. Thus, we suggest that identifying the determinants of weight control concerns and behaviors in future research is essential to analyze the mechanism through which weight control concerns and behaviors increase the likelihood of eating disorder symptoms, independent of actual weight. Moreover, research is needed on the association of weight control concerns and behaviors with other less extreme mental health outcomes.

## Abbreviations

BMI: body mass index; EDI: eating disorder inventory; OR: odds ratio; CI: confidence interval; DT: drive for thinness; B: bulimia; BD: body dissatisfaction; LSE: low self-esteem; ID: interoceptive deficits; P: perfectionism; MF: maturity fears

## Competing interests

The authors declare that they have no competing interests.

## Authors' contributions

YF and YL performed the statistical analyses, interpreted the results, wrote the manuscript and made substantial contributions. GM, YL, AL and XH were involved in the project design, questionnaire development and data assessment. GM was the principle investigator of present project. GM and GX supervised the progress of the study and revised the manuscript critically. All authors have read and approved the final version.

## Pre-publication history

The pre-publication history for this paper can be accessed here:

http://www.biomedcentral.com/1471-2458/10/314/prepub
